# Investigating the Impact of Carboxylated Polystyrene Nanoplastics in the Liver Using Cell Lines and Precision‐Cut Liver Slices

**DOI:** 10.1111/liv.70748

**Published:** 2026-06-14

**Authors:** Namrata Pandey, Paula Boeira, Nathaniel J. Clark, Eleanor T. Walker, Somaiah Aroori, Jemimah Denson, Richard C. Thompson, Matthew E. Cramp, Shilpa Chokshi, Ashwin Dhanda

**Affiliations:** ^1^ Centre of Environmental Hepatology, Peninsula Medical School, Faculty of Health University of Plymouth Plymouth England; ^2^ School of Health Professions, Faculty of Health University of Plymouth Plymouth England; ^3^ School of Biomedical Sciences, Faculty of Health University of Plymouth Plymouth England; ^4^ South West Liver Unit University Hospitals Plymouth NHS Trust Plymouth England; ^5^ School of Biological and Marine Sciences, Faculty of Science and Engineering University of Plymouth Plymouth England; ^6^ Roger Williams Institute of Liver Studies, Faculty of Medicine King's College London England

**Keywords:** hepatic cell lines, hepatotoxicity, human‐precision cut liver slices, nanoplastic, polystyrene

## Abstract

**Background and Aims:**

Increasing reports of plastic accumulation in human tissue have raised concerns about potential adverse health outcomes. Evidence of negative effects of nanoplastics is heterogeneous and provides limited insights into the underlying pathogenic toxicity mechanisms in humans.

**Methods:**

In the present study, carboxylate‐modified fluorescently labelled polystyrene nanoparticles (PS NPs) were used to investigate uptake and cytotoxicity in three different hepatic models of varying complexity, including HepG2 cells, IHH cells, and human precision‐cut liver slices (hPCLS).

**Results:**

The results show model‐ and dose‐dependent effects on hepatocytes. 74.2% ± 13.4% of the IHH cells showed PS NPs uptake at 0.1 μg/mL, which is considerably lower than the estimated plastic concentration in human blood (1.8–4.7 μg/mL). The viability of IHH cells decreased to 10.6% ± 9.1% after exposure to 100 μg/mL for 48 h. Early signs of hepatic injury were found in hPCLS at high concentrations. No changes were observed in the redox state and mitochondrial respiratory parameters of HepG2 cells after exposure.

**Conclusion:**

The PS NPs exposure experiments show uptake across all three hepatic models and toxic effects in IHH cells and hPCLS. Overall, the study highlights the need for physiologically relevant human tissue models to understand the impact of nanoplastic pollution on human health.

## Key Points

- PS NPs accumulate in hepaocytes even at low exposure levels.
- The effect of PS NPs on hepatocytes depends on the model and dose.
- High concentrations of PS NPs caused toxicity in IHH cells and the hPCLS model.

## Lay Summary

Particles of plastic described as microplastics (MP) and nanoplastics (NP) are accumulating in the environment from a range of sources, including the fragmentation of large plastic items during use and after disposal. NPs are invisible to the naked eye and can enter the human body by consuming food and drinks and breathing air containing these particles. Once inside the body, NPs can reach and accumulate in the organs and body tissues. This study used three models of the liver to test what happens after exposure to polystyrene NPs. It shows that polystyrene NPs can accumulate in the liver cells even at low exposure levels and can also cause damage and death of liver cells at higher levels. The study adds to the scientific evidence of harmful effects of NPs on human health, the environment, and economies and pushes for policies towards a sustainable future.

**Abbreviations**
7‐AAD: 7‐amino‐actinomycin D
ALT: alanine aminotransferase
AST: aspartate aminotransferase
DAPI 4′: 6‐diamidino‐2‐phenylindole
DCFDA 2′: 7′‐dichlorofluorescein diacetate
ECR: extracellular acidification rate
FCCP: carbonyl cyanide 4‐(trifluoromethoxy)phenylhydrazone
hPCLS: human precision‐cut liver slices
IHH: immortalised human hepatocytes
LDH: lactate dehydrogenase
NP: nanoplastic
OCR: oxygen consumption rate
PFA: paraformaldehyde
PS NPs: polystyrene nanoparticles
ROS: reactive oxygen species

## 1 Introduction

Micro and Nano‐plastic pollution is a pervasive environmental challenge that poses a credible concern to human health [1]. Humans are exposed to these particles via inhalation of aerosolised particles, ingestion of contaminated food and water, and, to a lesser degree, from dermal exposure [2]. Upon entering the human body, nanoplastics (NP, size < 1 μm) can enter the circulatory system, cross the endothelium and accumulate in various organs. Studies have reported plastics in different human organ systems and tissues [3, 4, 5, 6, 7, 8, 9, 10, 11], raising concerns that systemic exposure may act as a driver of organ‐specific pathology. Existing toxicological literature on model organisms suggests that plastics and their associated chemicals cause toxicity, but evidence in human studies is lacking [12]. Biodistribution studies performed in animal models have highlighted that polystyrene NP of size 80 nm can reach the circulatory system within 10 min of oral dose administration [13] and 50 nm polystyrene NP can accumulate in the mesenteric lymph node, spleen and liver, indicating translocation across the epithelial barrier [14].

The multifaceted role of the liver in the digestive and circulatory systems makes it highly vulnerable to plastic exposure via the gut‐liver axis. This exposure may be exacerbated in individuals with pre‐existing health conditions. Horvatitis et al. reported higher levels of microplastics in liver tissue from people with cirrhosis (8.3 ± 3.2 particles/g) compared to normal liver tissue (1.0 ± 0.7 particles/g) [15]. Yan et al. reported a higher concentration of microplastics in stool samples from patients with inflammatory bowel disease (41.8 items/g) compared to healthy individuals (28.0 items/g) [16]. These observations raise the question of whether plastic accumulation is a *cause* or a *consequence* of the rising global liver disease burden.

Recently, there has been a focus on NP due to their higher bioavailability and greater propensity for accumulating in the liver [17]. Most studies have utilised commercially available pristine polystyrene nanoparticles (PS NPs) for exposure experiments on cell culture and animal models. Findings indicate accumulation in hepatocytes, which can lead to cell membrane damage, oxidative stress, inflammatory response, irregularities in energy production, lysosomal dysfunction and autophagy. The existing literature on the adverse effects of NP is substantial; however, reports on hepatotoxicity of PS NP are variable [18, 19, 20, 21]. Despite this, oxidative stress is the most commonly observed mechanism of cytotoxicity. PS NP exposure increases the production of reactive oxygen species (ROS), disrupts the redox state, exacerbates NF‐κB‐mediated inflammation and mitochondrial damage, and drives cells towards apoptosis [20, 22]. Variations regarding the toxicity of NP on the liver may also be due to the limitations of the models utilised. Commonly used hepatoma cell lines, such as HepG2, are cancerous in origin and proliferate rapidly, accumulating genetic mutations and decreasing toxicity predictability [23].

The present study aimed to investigate the cellular uptake and physiological impact of carboxylate‐modified PS NPs on three human hepatic models of increasing complexity: hepatocarcinoma HepG2 cells, non‐cancerous immortalised hepatocytes (IHH), and human precision‐cut liver slices (hPCLS). The three‐dimensional liver slices conserve the native architecture, key liver functions for up to 5 days ex vivo and enable the study of pathophysiology in the tissue‐specific microenvironment [24, 25]. In addition, hPCLS also aligns with the FDA Modernisation Act 2.0, which promotes the transition away from mandatory animal testing towards more human‐relevant models.

The present study is the first to employ the normal human liver tissue‐derived hPCLS model along with two immortalised hepatic cell lines to comparatively and comprehensively understand the effects of commercially available NP on physiologically relevant hepatic models. Studies have reported polystyrene accumulation in human liver tissue [7, 15], and environmental weathering can introduce carbonyl and carboxyl functional groups on polystyrene, making them carboxylated [26]. The carboxylated PS NPs served as a relevant proxy for aged environmental plastics encountered by humans. The use of fluorescently labelled PS NPs provided the analytical advantages of visualisation and quantification of cellular uptake with confocal microscopy and flow cytometry, respectively. The cytotoxic effect of PS NPs exposure after 24 and 48 h was analysed through cell viability staining and quantification of cell injury markers. The mechanism of PS NPs cytotoxicity was investigated in HepG2 cells by estimating the production of ROS after 1, 4 and 24 h of exposure and assessing the bioenergetic capacity of the mitochondria using the extracellular flux Seahorse assay. Overall, the study provides insight into the dose‐ and model‐dependent variability of NP exposure in the three hepatic models.

## 2 Materials and Methods

### 2.1 Characterisation of Carboxylate‐Modified Fluorescently Labelled 20 and 30 Nm Polystyrene Nanoparticles (PS NPs)

Red fluorescently labelled 20 nm (2% solids; ThermoFisher, F8786) and green fluorescently labelled 30 nm (2.5% solids; Merck, L5155‐1ML) carboxylate‐modified polystyrene nanoparticles (PS NPs) were used in the study. The stock suspensions were diluted in ultrapure water for characterisation. A drop of the aqueous suspension was dried on the copper grid and imaged using a JEOL 1400 transmission electron microscope (JEOL Ltd., Tokyo, Japan). Additionally, the stock suspension was analysed using NanoSight Pro (Malvern Panalytical, Worcestershire, UK) to determine the hydrodynamic diameter of the particles. The fluorescence from the 30‐nm particles could be recorded using FACSAria II (BD Biosciences, San Jose, US) at excitation 480 nm and emission 500 nm (Figure S1). The fluorescent signal from 20 nm PS NPs could be visualised using the SP8 Lightning confocal microscope (Leica Microsystems, Wetzlar, Germany) at excitation 580 nm and emission 605 nm (Figure S2). The polymeric composition of 30‐nm PS NPs was confirmed using pyrolysis gas chromatography and mass spectrometry (Figure S3).

### 2.2 Ethical Approval for Human Tissue

Human liver tissue was obtained from patients following partial hepatectomy for metastatic colorectal cancer at the University Hospitals Plymouth NHS Trust (Plymouth, UK), who had consented to the use of excess liver tissue (resection surplus) for research. All participants were anonymised with a unique code and with the reference list held in a password‐protected file on a secure server within University Hospitals Plymouth NHS Trust, with access only to the investigators. The NHS Health Research Authority London—Hampstead Research Ethics Committee (reference number 15/LO/0948) granted ethical approval for the study.

### 2.3 Human Precision‐Cut Liver Slices

Liver tissue was obtained from the distal portion of the resected specimen, immediately placed in ice‐cold Custodiol HTK (Histidine‐Tryptophan‐Ketoglutarate) organ preservation solution at the time of collection and transported to the laboratory for precision‐cut liver slice preparation.

Liver slices were generated in cold Krebs–Henseleit buffer (2.5 mM CaCl_2_, 118 mM NaCl, 5 mM KCl, 1.1 mM MgSO_4_, 1.2 mM KH_2_PO_4_, 25 mM NaHCO_3_, 25 mM D‐glucose, and 9 mM HEPES; pH 7.42) saturated with carbogen (95% O_2_/5% CO_2_), using a Krumdieck tissue slicer (Alabama R&D, Munford, AL, USA), as described by de Graaf et al. [27]. hPCLS were cut to a diameter of 5 mm and a thickness of 250 μm. Freshly prepared slices were cultured individually in 12‐well plates, each containing 1.5 mL of Williams E Medium supplemented with penicillin (100 IU/mL), streptomycin (100 μg/mL), 2 mM glutamine, ITS (10 mg/L insulin, 5.5 mg/L transferrin, 6.7 μg/L sodium selenite), 1 nM epidermal growth factor, 5% human AB serum, 100 nM glucagon, and 1 μM corticosterone. Plates were placed on an orbital shaker (70 rpm) inside an incubator at 37°C and maintained in carbogen‐filled chambers (95% O_2_/5% CO_2_) under humidified conditions. Following a 2‐h recovery period, hPCLS were transferred to fresh medium in new plates and incubated for an additional 24 h prior to experimental use.

### 2.4 Cell Line Cultures

HepG2 cell line was purchased from Sigma Aldrich (Lot No. 22E001) and cultured in T‐25 flasks in growth media containing DMEM media (supplemented with 4.5 g/L glucose, L‐glutamine, and sodium pyruvate), 10% *v*/*v* FBS and 1% *v*/*v* Pen/Strep at 37°C and 5% humidified CO_2_ conditions. The media was changed every 2 days. The IHH cells were cultured in DMEM/F12 media, 10% *v*/*v* FBS, 1% *v*/*v* Pen/Strep, 0.1% L‐glutamine, 20 mU/mL insulin and 50 nM/mL dexamethasone at 37°C and 5% humidified CO_2_ conditions. The media was changed after 24 h of passaging and every 2 days for maintenance. After 70% confluency, the cells were seeded in 24‐well cell culture plates and maintained for experiments.

### 2.5 Cellular Uptake of PS NPs and Cell Viability in Hepatic Models

Cellular uptake of fluorescently labelled PS NPs was visualised using confocal microscopy and quantified with flow cytometry. HepG2 (*n* = 3) and IHH (*n* = 3) cells were seeded at a density of 2 × 10^5^ cells/well in 24‐well culture plates on sterile 13‐mm diameter glass coverslips in each well and incubated overnight. The particle stock suspension was diluted using fresh culture media to obtain the desired exposure concentrations for the experiments. After incubation, the growth media was aspirated and the cells were exposed to 20 nm PS NPs at concentrations of 0.1, 1, 5 and 10 μg/mL. After 24 h, the exposure media was aspirated, and the cells were washed twice with sterile PBS. The cells were fixed with 4% paraformaldehyde (PFA, ThermoFisher, Cat no. J19943‐K2) for 20 min and stained with 0.4% 4′,6‐diamidino‐2‐phenylindole (DAPI, ThermoFisher, Cat no. 62248) for 20 min in the dark at room temperature. The coverslips were then mounted using prolonged diamond mounting media (ThermoFisher, Cat no. P36965) and observed under the Leica SP8 confocal microscope at 63× objective under oil immersion. Further, the quantification of PS NPs uptake in the cell lines was determined by seeding 2 × 10^5^ cells/well in 24‐well culture plates and exposing them to increasing concentrations of 30 nm PS NPs at 0.1, 1, 10 and 100 μg/mL (*n* = 3). After 24 and 48 h, the cells were trypsinised, washed with FACS buffer (500 mL sterile PBS, 2% FBS and 0.4% 0.5 M EDTA), stained with 7‐amino‐actinomycin D (7‐AAD, Invitrogen, Cat no. 00‐6993‐50) for 5 min at room temperature and analysed using FACSAria II at 485/520 nm. The cell viability stain, 7‐AAD, did not give a distinct positive signal for the HepG2 positive control cells; therefore, viability was determined using forward and side scatter plots from flow cytometry analysis (Figure S4).

For the hPCLS experiments, after 24 h in culture, the slices were transferred to 12‐well culture plates with increasing concentrations of 30 nm PS NPs exposure medium for 24 h (*n* = 5). The prepared slices were placed on an orbital shaker (70 rpm) inside an incubator at 37°C and maintained in carbogen‐filled chambers (95% O_2_/5% CO_2_) under humidified conditions. The exposure concentrations were 10, 25, 50 and 100 μg/mL. After 24 h exposure, slices were washed with PBS and transferred to new plates containing 1 mL of liver digest medium (Gibco) for 30 min for dissociation of viable liver cells. After digestion, liver cells were collected in FACS buffer. Cells were then stained with 7‐AAD for 5 min at room temperature to assess cell death. Samples were analysed using FACSAria II, followed by fluorescence microscopy using EVOS M5000 (Invitrogen, Waltham, USA) (Figure S5).

### 2.6 Hepatic Injury Markers in HepG2, IHH and hPCLS

HepG2 (*n* = 3) and IHH (*n* = 3) cells were exposed to 30 nm PS NPs in serum‐free media for 24 and 48 h. For hPCLS, complete media with 30 nm PS NPs was used (*n* = 5). The culture media from the three models were collected and centrifuged at 1800 rpm for 5 min to obtain the supernatant. Ten microlitres of the supernatant was used to measure lactate dehydrogenase (LDH), aspartate aminotransferase (AST), and alanine aminotransferase (ALT) levels, using the Fujifilm DRI‐CHEM NX600 (Fujifilm, Bedford, UK) to assess cellular toxicity. The instrument quantifies enzymes and biochemicals using a colorimetric method and presents results in international units/L (U/L).

### 2.7 Estimation of Reactive Oxygen Species Generation

HepG2 cells were seeded at a density of 80 000 cells/well in 24‐well cell culture plates and incubated overnight at 37°C under humidified 5% CO_2_ conditions. Cells were then exposed to 0.1, 1, 10 and 100 μg/mL 30 nm PS NPs for 1, 4 and 24 h (*n* = 6). After the exposure, wells were washed with sterile PBS and incubated with 10 μM 2′,7′‐Dichlorofluorescein diacetate (DCFDA, Sigma‐Aldrich Cat no. D6883‐50MG) for 30 min at 37°C in the dark. Cells were then trypsinised, washed once with FACS buffer, resuspended in 400 μL of FACS buffer, and analysed using FACSAria II. Shorter exposure durations were studied to capture the immediate toxicity of PS NPs exposure. A 2.5 mg/mL FeSO_4_.7H_2_O solution exposure for 4 h was used as a positive control (Figure S6) [28]. The results were normalised to the positive control and presented as the percentage normalised fluorescent intensity.

### 2.8 Measurement of Mitochondrial Bioenergetics

Cell culture density for HepG2 cells and the concentrations of the uncouplers/inhibitors involved in the experiment were optimised before running the experiments (Table S1). Thirty thousand cells were seeded in an XFe96 Seahorse cell culture plate (excluding the background wells) and maintained at 37°C with 5% CO_2_ humidified conditions overnight. Cells were exposed to 80 μL of 20 nm PS NPs exposure media in concentrations of 0.1, 1, 10 and 100 μg/mL for 24 h (*n* = 5). One day prior to performing the assay, the Seahorse XF sensor cartridge was hydrated with Seahorse XF calibrant solutions (Agilent, Part no. 100840‐00010023001) by filling 180 μL/well. The cartridge was incubated in a CO_2_‐free incubator maintained at 37°C overnight. Post‐exposure, the media was aspirated, and wells were washed 3 times with XF assay media (Agilent, Part no. 103575‐10020623002) supplemented with 2 mM glutamine, 1 mM pyruvate and 10 mM glucose. The XF cell culture plate was then incubated in a CO_2_‐free incubator maintained at 37°C for 45 min. The XF inhibitor/uncoupler plate was prepared using the Agilent Seahorse Cell Mito Stress kit (Agilent, Part no. 103015‐100) at optimised concentrations of oligomycin, carbonyl cyanide 4‐(trifluoromethoxy)phenylhydrazone (FCCP) and rotenone/antimycin A (Figure S7). The drugs were administered in the same sequence. Oxygen consumption rate (OCR) and extracellular acidification rate (ECAR) were measured by Agilent Seahorse XFe96 Analyser (Agilent, Santa Clara, USA) according to the manufacturer's instructions. The OCR data was normalised to total protein content per well, estimated using the BCA assay.

### 2.9 Quality Control and Assurance

The inadvertent contamination of MP/NP in all experimental work was minimised by practising strict QA and QC measures. Personal protective equipment, including 100% cotton laboratory coats and nitrile gloves, was worn throughout all procedures. All experiments were performed within a Class II microbiological safety cabinet to limit airborne particulate contamination. All experiments included three or more technical replicates, including the control cultures, which were handled identically to exposure groups but without the addition of nanoplastics. Tissue culture media were prepared fresh on the day of each experiment and filtered using 0.25‐μM sterile syringe filters to maintain sterility and reduce the introduction of extraneous particulates. These procedures were applied across all experimental conditions.

### 2.10 Data Analysis

All experiments were performed in three or more independent replicates. All flow cytometry raw data were analysed in FlowJo v. 10.9.0. The Seahorse XFe96 data files were processed and analysed in Wave 2.6.3. Data analysis was performed on experimental data with three or more replicates using GraphPad Prism software version 8.0.2. The values are reported as Mean ± SEM. Comparative statistical analysis was not performed due to the small number of replicates per group.

## 3 Results

### 3.1 Characterisation of PS NPs

**FIGURE 1:**
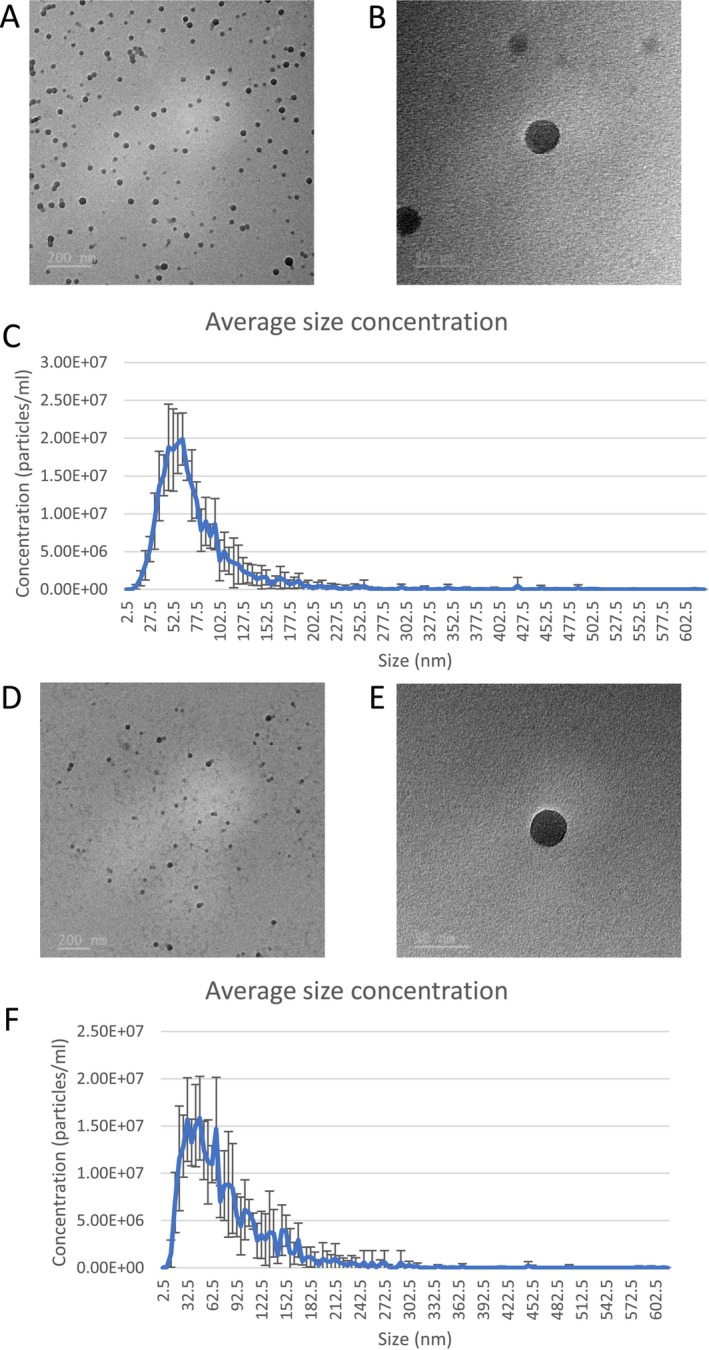
Particle characterisation of PS NPs. (A, B) TEM images at 20 000× and 100 000× magnification, and (C) size distribution histogram of 20 nm PS NPs; (D, E) TEM images at 15 000× and 100 000× magnification and (F) size distribution histogram of 30 nm PS NPs.

The TEM images show the uniform spherical shape of the 20 and 30 nm PS NPs, as per the manufacturer's specifications (Figure 1). The NanoSight Pro was used to determine the hydrodynamic diameter of the 20 and 30 nm PS NPs in ultrapure water. The modal size distribution of 5 replicates for 20 nm and 30 nm PS NPs was in the range of 47.5–62.5 nm and 32.5–67.5 nm, respectively.

### 3.2 PS NPs Uptake in HepG2, IHH and hPCLS Model Is Dose‐Dependent

**FIGURE 2:**
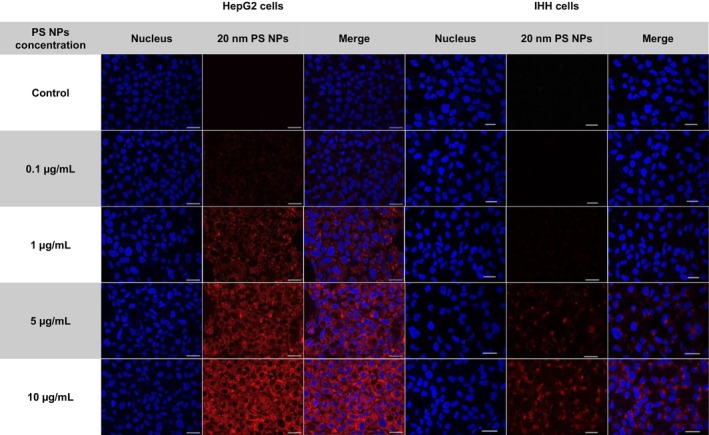
Confocal microscopy images of HepG2 (left panel) and IHH (right panel) cell lines exposed to 20 nm PS NPs at 63×, scale bar represents 25 μm.

PS NPs uptake in HepG2 and IHH cell lines was performed using confocal microscopy. An increase in the fluorescent signal was observed in HepG2 cells exposed to 20‐nm red fluorescent PS NPs at concentrations of 1, 5 and 10 μg/mL, with no distinct difference in the cellular distribution of the particles and some agglomeration within the cells (Figure 2). Similar morphological observations were made in IHH cells, except that distinct fluorescent signals were observed only at concentrations of 5 and 10 μg/mL.

**FIGURE 3:**
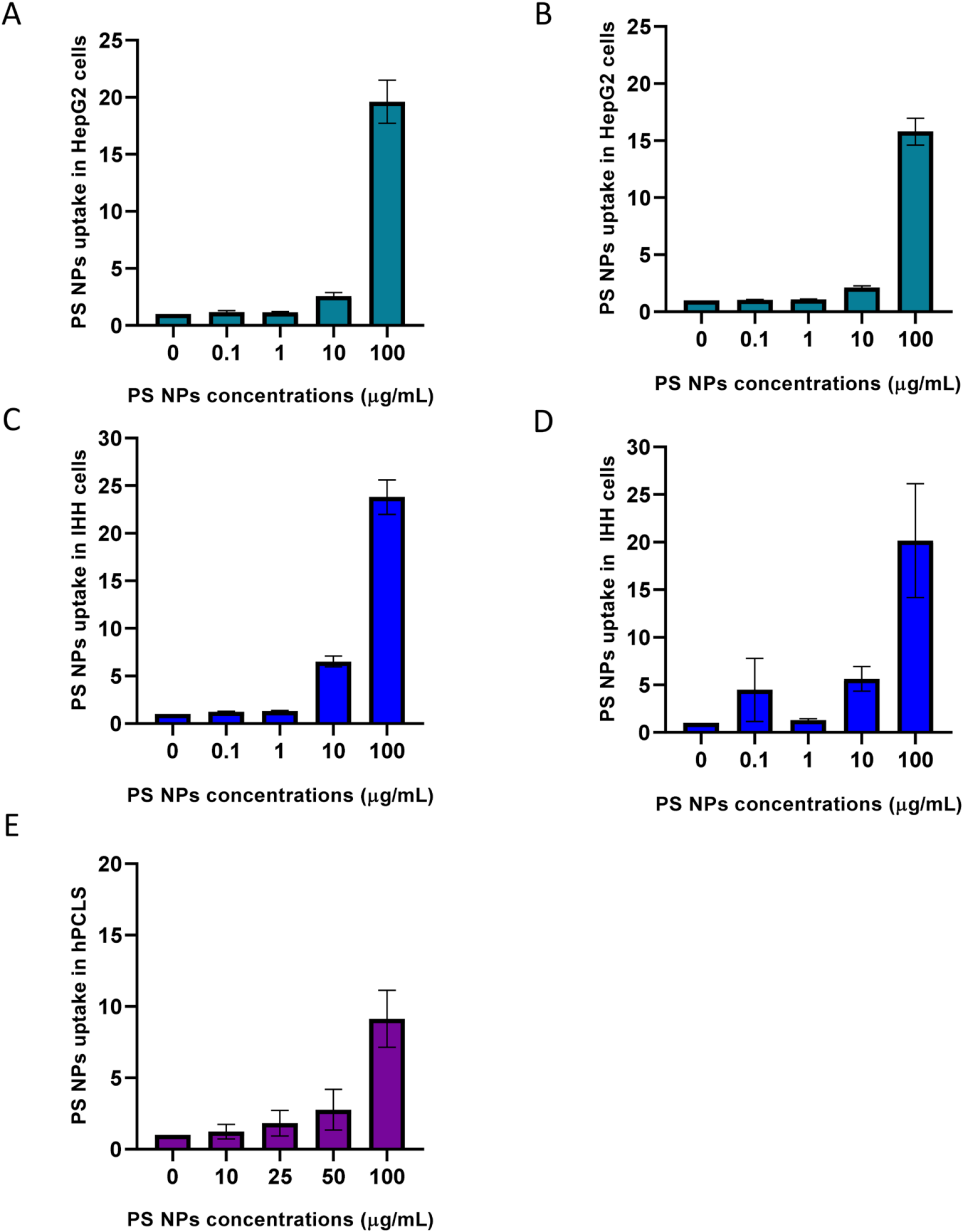
Quantification of PS NPs uptake in HepG2 cells after (A) 24 h (*n* = 3) and (B) 48 h (*n* = 3); IHH cells after (C) 24 h (*n* = 3) and (D) 48 h (*n* = 3); (E) hPCLS after 24 h exposure (*n* = 5).

In addition to this, PS NPs uptake in HepG2, IHH and hPCLS was quantified using flow cytometry (Figure 3). The results are consistent with confocal images for HepG2 and IHH, showing a dose‐dependent uptake of 30 nm PS NPs after 24 and 48 h. The fluorescence signal from cells exposed to 10 and 100 μg/mL PS NPs for 24 h was 2.5 ± 0.3 and 19.6 ± 1.8 times higher in HepG2, and 6.5 ± 0.5 and 23.8 ± 1.8 times higher in IHH, compared to their respective control groups. PS NPs uptake in the hPCLS model demonstrated a dose‐dependent response (*n* = 5; baseline characteristics of the tissue donors are listed in Table S2). At an exposure concentration of 100 μg/mL, uptake in hPCLS isolated cells was 9.1 ± 2 times more than that of the control.

**FIGURE 4:**
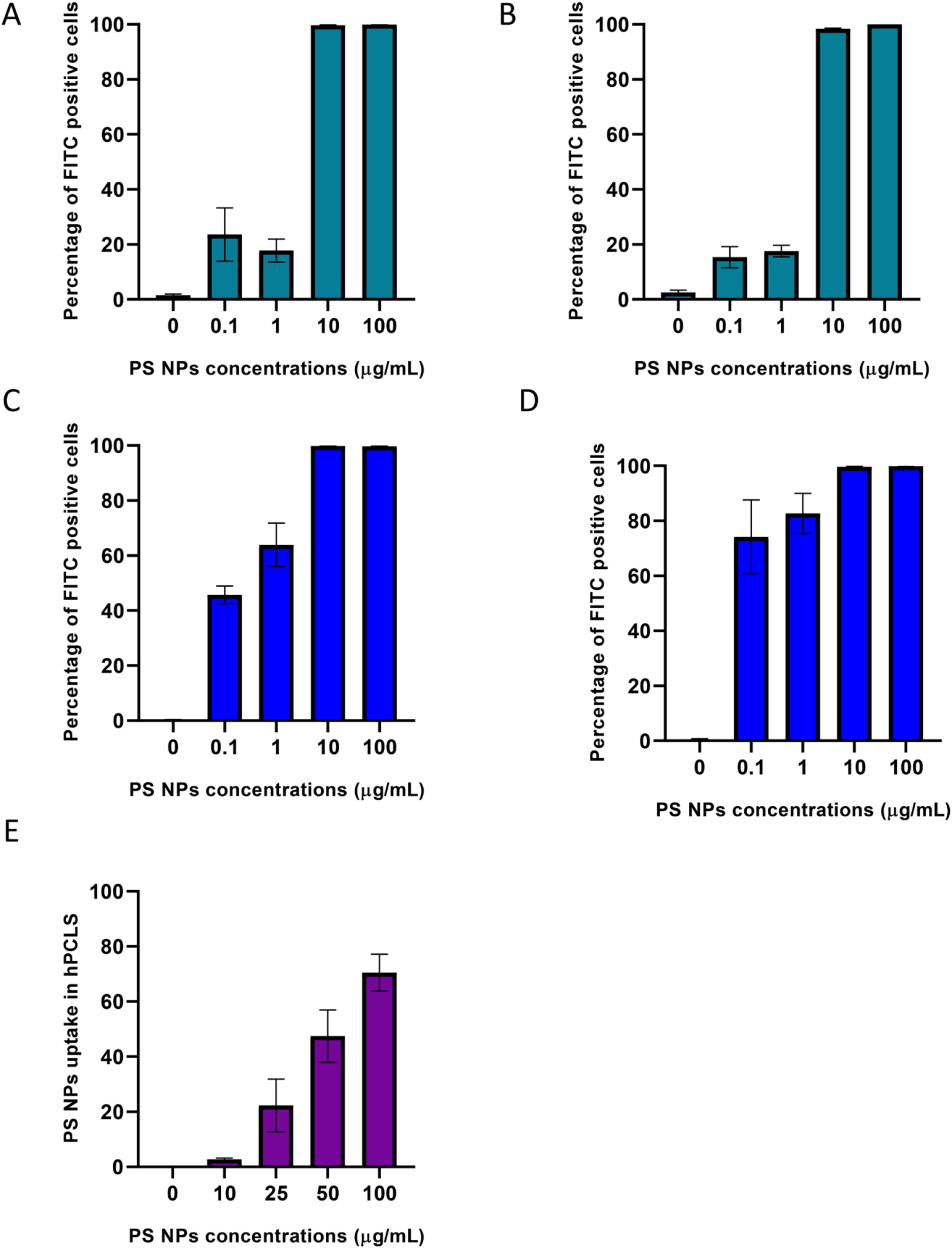
Percent cellular uptake of PS NPs in HepG2 cells after (A) 24 h (*n* = 3) and (B) 48 h (*n* = 3); IHH cells after (C) 24 h (*n* = 3) and (D) 48 h (*n* = 3); (E) hPCLS after 24 h exposure (*n* = 5).

The percentage of cells with PS NPs uptake showed a dose‐dependent response in the hepatic models (Figure 4). In HepG2 cells, PS NPs uptake reached saturation at concentrations 10 and 100 μg/mL, with 99.7% ± 0.05% and 100% ± 0% cells, respectively, while only 23.6% ± 9.7% cells showed uptake at 0.1 μg/mL, after 24 h of exposure. In IHH cells, 99.8% ± 0.1% and 99.7% ± 0.1% cells showed uptake at 10 and 100 μg/mL, respectively, after 24 h of exposure. PS NPs uptake reached saturation in IHH cells after 48 h, with 74.2% ± 13.4% cells at 0.1 μg/mL and 99.8% ± 0.13% cells at 100 μg/mL. In hPCLS, cellular PS NPs uptake was observed in 70.5% ± 6.7% cells at 100 μg/mL, which is similar to that in IHH cells.

### 3.3 Effect of PS NPs Exposure on Cell Viability and Hepatic Injury Markers

**FIGURE 5:**
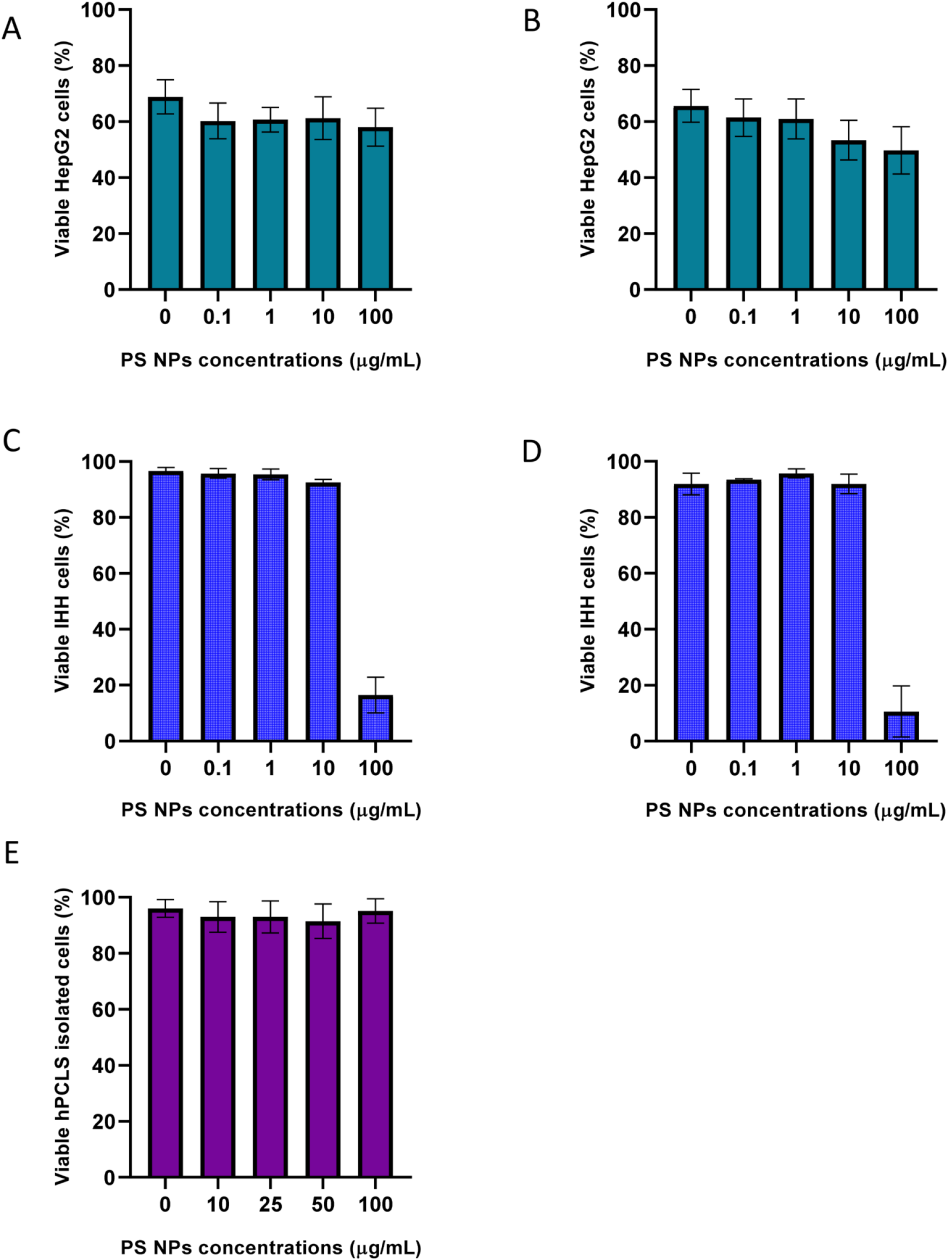
Effect of PS NPs on cell viability of HepG2 cells after (A) 24 h (*n* = 3) and (B) 48 h (*n* = 3); IHH cells after (C) 24 h (*n* = 3) and (D) 48 h (*n* = 3); (E) hPCLS after 24 h exposure (*n* = 5).

**FIGURE 6:**
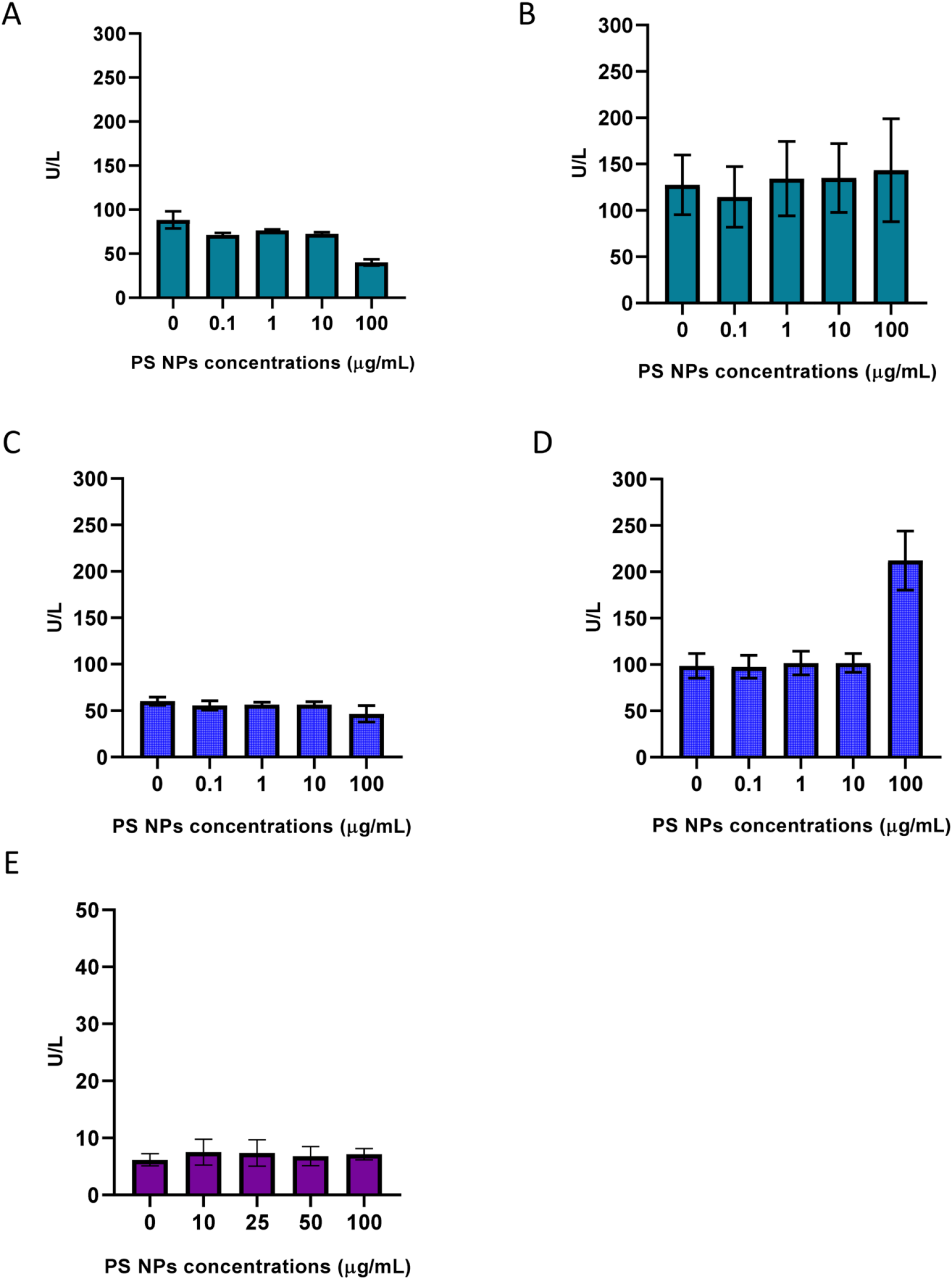
LDH levels in the media after PS NPs exposure in HepG2 cells after (A) 24 h and (B) 48 h; IHH cells after (C) 24 h and (D) 48 h; (E) hPCLS after 24 h exposure.

**FIGURE 7:**
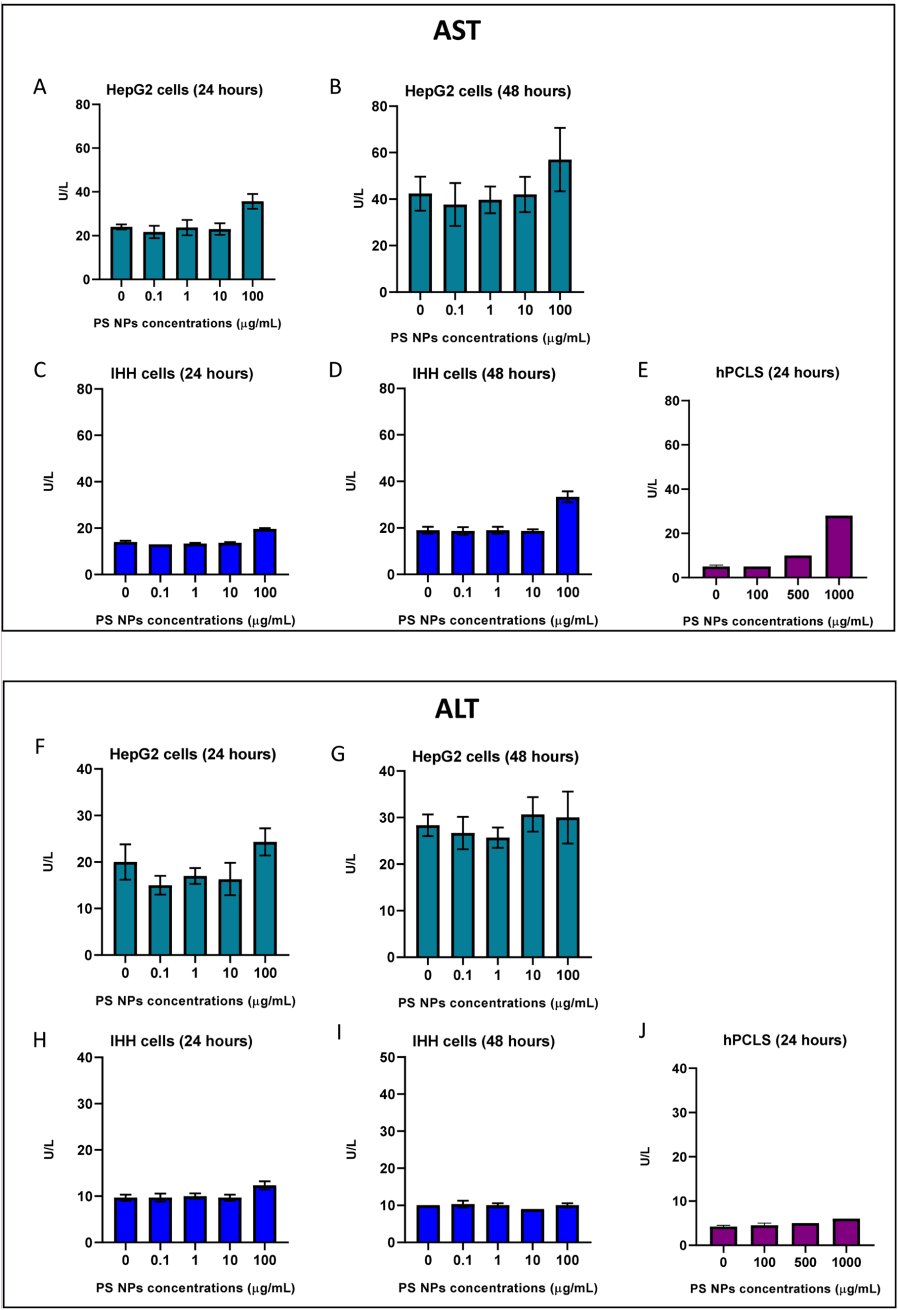
Analysis of cellular injury markers in hepatic models exposed to PS NPs. (Top panel) AST levels in media from HepG2 cells after (A) 24 h (*n* = 3) and (B) 48 h (*n* = 3), IHH cells (C) 24 h (*n* = 3) and (D) 48 h (*n* = 3), (E) hPCLS after 24 h (*n* = 1 for 500 and 1000 μg/mL). (Bottom panel) ALT levels in the media from HepG2 cells after (F) 24 h (*n* = 3) and (G) 48 h (*n* = 3), IHH cells (H) 24 h (*n* = 3) and (I) 48 h (*n* = 3), (J) hPCLS after 24 h (*n* = 1 for 500 and 1000 μg/mL).

Cell viability of HepG2, IHH and hPCLS was analysed after 24 and 48 h of PS NPs exposure to explore the cytotoxic effects of PS NP on the hepatic models. The results show no comparable difference in the viability of HepG2 cells and hPCLS exposed to PS NPs, even at the highest concentrations. In IHH cells, cell viability decreased after exposure to the highest PS NPs concentration. After 24 h, the percentage of viable cells was 96.6% ± 1.2% in the control group and only 16.5% ± 6.4% in the 100 μg/mL group. After 48 h, the percentage of viable cells decreased to 10.6% ± 9.1% in the 100 μg/mL group (Figure 5C,D). Cell viability was also confirmed by measuring the LDH levels in the culture media after exposure. LDH levels of IHH cells after 48 h exposure at 100 μg/mL were 212.3 ± 31.8 U/L compared to 98.7 ± 13.3 U/L in the control group (Figure 6D). This shows cellular damage and apoptosis in the IHH cells after PS NPs exposure. The levels of AST and ALT enzymes released in the media post 24 and 48 h PS NPs exposure in the three hepatic models were analysed. The AST levels of IHH cells exposed to 100 μg/mL PS NPs for 48 h were 33.3 ± 2.3 U/L compared to 19.0 ± 1.5 U/L in the control group (Figure 7D). In hPCLS, AST levels in the control group were 5 ± 0.7 U/L, which increased to 10 and 28 U/L post 24 h exposure to PS NPs concentrations of 500 and 1000 μg/mL (Figure 7E).

### 3.4 Assessment of Alternative Toxicity Endpoints in HepG2 Cells

**FIGURE 8:**
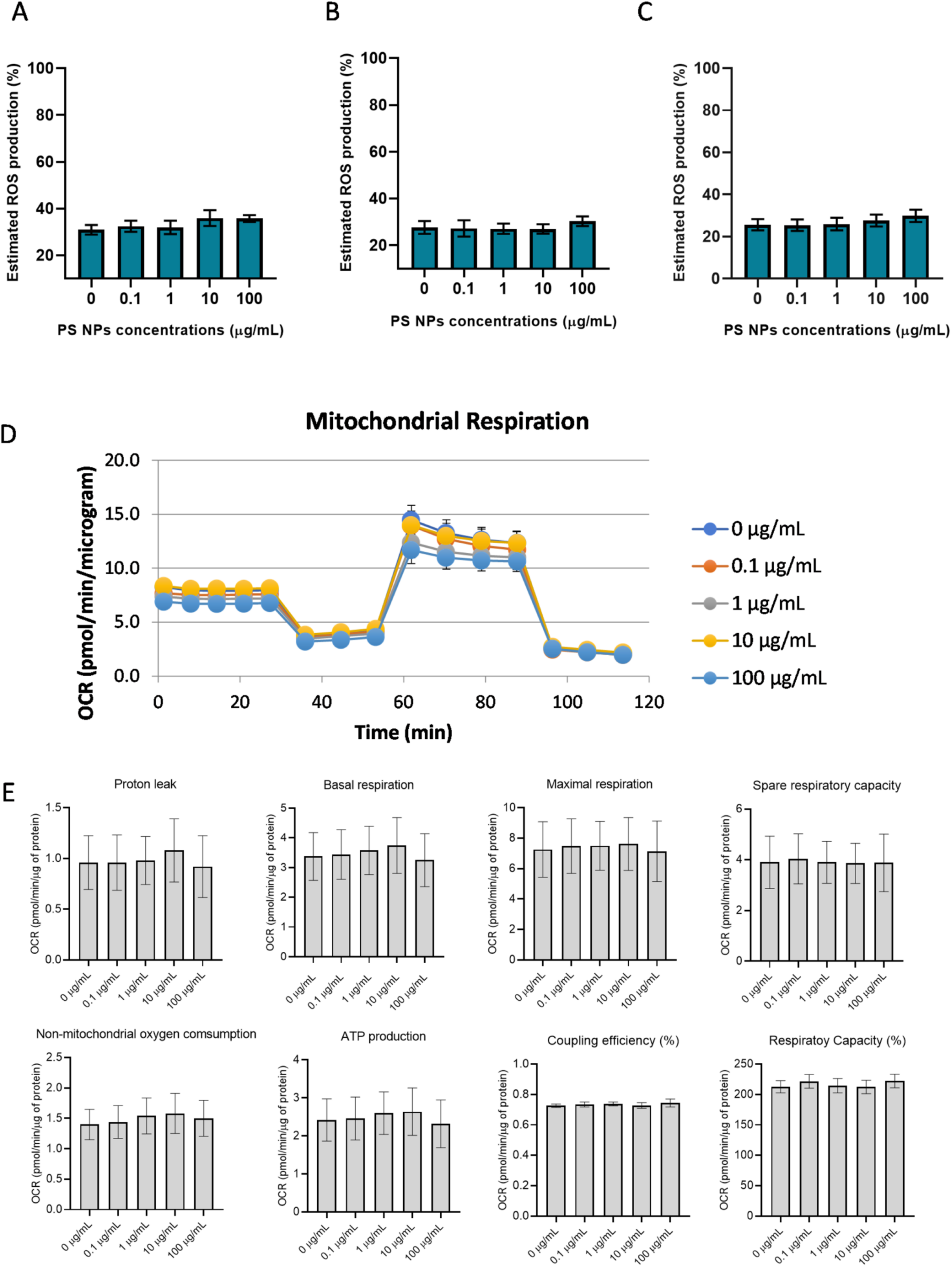
Physiological effect of PS NPs exposure on HepG2 cells. Estimated ROS levels in HepG2 cells after (A) 1 h (*n* = 6), (B) 4 h (*n* = 6) and (C) 24 h (*n* = 3) of exposure. Analysis of (D) OCR and (E) mitochondrial respiratory parameters in HepG2 cells after 24 h (*n* = 5).

Given the minimal overt cytotoxicity observed in HepG2 hepatoma cells following exposure to PS NPs, subtle toxicological responses were further investigated. Specifically, the markers of oxidative stress and mitochondrial dysfunction were assessed to determine whether sub‐lethal cellular stress pathways were activated despite the absence of classical toxicity indicators. HepG2 cells were exposed to increasing concentrations of PS NPs particles for 1, 4 and 24 h. The data show a small increase in the ROS production at 100 μg/mL compared to the control at 1, 4 and 24 h of exposure (Figure 8A–C). The effects of acute PS NPs exposure on the mitochondrial respiratory capacity of HepG2 cells were studied using the Mito stress kit with Seahorse XFe96 Analyser. The results indicate a similar response in respiratory parameters in cells exposed to high concentrations of PS NPs for 24 h compared to the control (Figure 8D,E). These results demonstrate that acute PS NPs exposure does not disrupt the redox state and mitochondrial respiration in HepG2 cells.

## 4 Discussion

In the present study, the effects of carboxylate‐modified PS NPs were studied on HepG2, IHH, and hPCLS. All the hepatic models showed a dose‐dependent uptake of NP from 10 μg/mL. A decrease in cell viability and cell injury was observed in IHH at 100 μg/mL, and in hPCLS at 1000 μg/mL. Further examination of toxicity mechanisms in HepG2 cells showed no change in oxidative stress and mitochondrial respiration at the highest PS NPs concentration. The study shows that PS NP toxicity is dose and model‐dependent, and the use of physiologically relevant models is crucial to examine the underlying hepatic pathophysiology.

The uptake of PS NPs in HepG2, IHH and hPCLS was observed using confocal microscopy and quantified with flow cytometry in a wide concentration range. The higher percentage of PS NPs positive IHH cells at 0.1 μg/mL is noteworthy. This concentration is within the range of estimated total plastic concentration in the blood of healthy individuals, which ranges from 1.6 to 4.65, and 1.6 μg/mL [5, 6]. The saturation of IHH cells with PS NPs at the lowest concentration demonstrates the bioavailability of particles and their ability to enter and accumulate in normal human cells. While uptake in lower PS NP concentrations was not apparent in HepG2 and hPCLS, the cellular uptake was confirmed in concentrations above 10 μg/mL, with no effect on cell viability. In IHH cells, cell viability decreased by 90% after 48 h exposure to 100 μg/mL, which was confirmed by an increase in LDH levels in the media. The AST level in IHH and hPCLS media was higher at the highest PS NP concentrations compared to the control. Higher levels of AST in the media can be indicative of mitochondrial membrane damage and loss of cell integrity.

Since HepG2 cells showed no acute toxicity from PS NPs exposure, subtle physiological changes in the hepatoma cells were studied by analysing oxidative stress and mitochondrial respiration. The overall change in the redox state of the HepG2 cells after exposure to 20 nm PS NPs for 1, 4 and 24 h showed no difference from the control. The results are consistent with other studies in the literature [21, 29]. In addition, HepG2 cells did not exhibit any change in mitochondrial respiratory capacity after acute PS NPs exposure. Bioenergetics analysis to study NP toxicity has been explored in various cell lines; however, studies are limited and variable [30, 31, 32].

Several MP/NP exposure studies have explored cytotoxic effects in vitro and in vivo, but the mechanisms of hepatotoxicity at the subcellular level remain unknown. One plausible and widely accepted hepatotoxicity mechanism of PS NP uptake in cells is an imbalance in the redox state, driven by increased ROS production, which aggravates mitochondrial damage and cell apoptosis [20]. A study on the cytotoxic effects of functionalised 50 nm PS NPs reported that exposure of carboxylate‐modified PS NPs at a concentration of 100 μg/mL can exhibit stronger antioxidant ability in HepG2 cells compared to those exposed to aminated PS NPs [19]. Another study examining the spectrochemical cytotoxicity in HepG2 cells exposed to 50 nm amine, carboxylate, and unmodified PS NPs showed that carboxylated PS NPs can lead to changes in DNA expression levels and irreversible modifications to the secondary structure of proteins within the cells [33]. A study on THLE‐2, normal human liver cells, demonstrated that PS NPs exposure can disturb the metabolic profile of the cells and alter their chemosensitivity to xenobiotics [29].

NP toxicity can be influenced by a series of physicochemical parameters, such as size, shape, polymer characteristics, and surface modifications [34]. However, the difference in the physiology of variable cell types is a crucial factor in mechanism studies [35]. The hepatoma cell line can accumulate genetic modifications, which can decrease toxicity predictability and increase variability. This can explain the lack of any functional changes in the oxidative parameters and energy production in HepG2 cells after exposure to PS NP at high concentrations. To overcome this challenge, IHH cell lines were used in the study as they retain more liver‐specific functions and offer the advantage of working with normal hepatocytes. This can be appreciated with the observed difference in the uptake and cytotoxic effects of PS NPs between the two cell lines. Two‐dimensional in vitro models lack the structural integrity and native complexity of the liver. The use of hPCLS in this study provides a three‐dimensional physiologically relevant model to study NP exposure and demonstrates the accumulation of PS NPs in hPCLS at 10 μg/mL. The increased AST levels at the highest exposure concentration demonstrate early signs of hepatic injury.

This study has some limitations. The commercially available uniform carboxylate‐modified fluorescently labelled PS NPs used in this study are not representative of ecologically relevant NPs. In the environment, NPs exist in heterogeneous mixtures with other persistent pollutants and undergo weathering that causes a shift in the physicochemical properties, such as surface modifications and protein corona formation, which can affect NPs in vivo and influence the NP‐cell interactions. PS is the most widely used polymer in the literature, understating the potential cytotoxic effects of other common commercially used polymers. Exposure concentrations above 10 μg/mL are high and not representative of the systemic exposure of plastic in humans directly. However, they were required to elicit cell injury and study the impact of PS NP on physiologically relevant models. Studies exploring the response of NP exposure to disease burden challenge in models are also necessary. This study demonstrates the ability of hepatocytes to uptake carboxylate‐modified PS NPs. It emphasises the development and use of improved toxicity models to study the role of plastics in human health.

## 5 Conclusion

In conclusion, HepG2, IHH, and hPCLS exposed to 20 nm PS NPs for 24 and 48 h, all showed dose‐dependent accumulation, which for IHH occurred at concentrations similar to those previously reported in human blood. PS NP exposure decreased the cell viability and caused cellular injury to IHH cells and hPCLS at the highest exposure concentrations. No changes in the oxidative stress and mitochondrial respiration were observed in HepG2 cells after PS NP exposure. The contrasting results between the three models indicate a need to shift from the over‐reliance on routinely used two‐dimensional in vitro cell models to more physiologically relevant models that encapsulate the toxicity mechanisms of a highly variable pollutant like nanoplastics.

## Author Contributions

Experiments and drafting of the manuscript: N.P. and P.B. Study design, interpretation, and critical review of the manuscript: N.J.C., R.C.T., M.E.C., S.C. and A.D. Patient consent and sample acquisition: E.T.W., J.D. and S.A. All authors approved the final version of the article, including the authorship list.

## Funding

N.P. was supported by the PhD studentship award from the Faculty of Health, University of Plymouth.

## Ethics Statement

The authors confirm that ethical approval has been obtained for this study. All participants were anonymised using a unique code, and the reference list was held in a password‐protected file on a secure server to maintain confidentiality. The NHS Health Research Authority London—Hampstead Research Ethics Committee (reference number 15/LO/0948) granted ethical approval for the study.

## Consent

The authors confirm that written informed consent was obtained from all the participants prior to their inclusion in the study.

## Conflicts of Interest

The authors declare no conflicts of interest.

## Supporting information


**Figure S1:** Fluorescence spectra of green fluorescently labelled 30 nm polystyrene nanoplastics, excitation 480 nm and emission at 500 nm.
**Figure S2:** Fluorescence spectra of red fluorescently labelled 20 nm polystyrene nanoplastics, excitation at 580 nm and emission at 605 nm.
**Figure S3:** Extracted ion chromatogram (m/z 91, 104) of 30‐nm polystyrene nanoplastics.
**Figure S4:** Gating strategy for cell viability analysis in IHH and HepG2 cells.
**Figure S5:** Fluorescence microscopy images of cells isolated from liver slices showing PS NPs uptake in hPCLS.
**Figure S6:** 2.5 mg/mL FeSO4.10H2O was used as a positive control for DCFDA assay.
**Figure S7:** Seahorse XF Cell mito stress test profile, showing key parameters for mitochondrial function.
**Table S1:** Inhibitors/Uncoupler concentrations and measurement details for the Seahorse XFe96 assay.
**Table S2:** Baseline characteristics of liver tissue donors for precision‐cut liver slices preparation.

## Data Availability

The data that support the findings of this study are available from the corresponding author upon reasonable request.
