# Supplementary material for: Investigating the Impact of Carboxylated Polystyrene Nanoplastics in the Liver Using Cell Lines and Precision‐Cut Liver Slices
Source: Liver Int. 2026 Jun 14;46(7):e70748. doi: 10.1111/liv.70748 (PMC13266279; doi:10.1111/liv.70748)
Supplement: Supplementary file 1 — Figure S1: Fluorescence spectra of green fluorescently labelled 30 nm polystyrene nanoplastics, excitation 480 nm and emission at 500 nm. Figure S2: Fluorescence spectra of red fluorescently labelled 20 nm polystyrene nanoplastics, excitation at 580 nm and emission at 605 nm. Figure S3: Extracted ion chromatogram (m/z 91, 104) of 30‐nm polystyrene nanoplastics. Figure S4: Gating strategy for cell viability analysis in IHH and HepG2 cells. Figure S5: Fluorescence microscopy images of cells isolated from liver slices showing PS NPs uptake in hPCLS. Figure S6: 2.5 mg/mL FeSO4.10H2O was used as a positive control for DCFDA assay. Figure S7: Seahorse XF Cell mito stress test profile, showing key parameters for mitochondrial function. Table S1: Inhibitors/Uncoupler concentrations and measurement details for the Seahorse XFe96 assay. Table S2: Baseline characteristics of liver tissue donors for precision‐cut liver slices preparation. [file LIV-46-0-s001.docx]

**
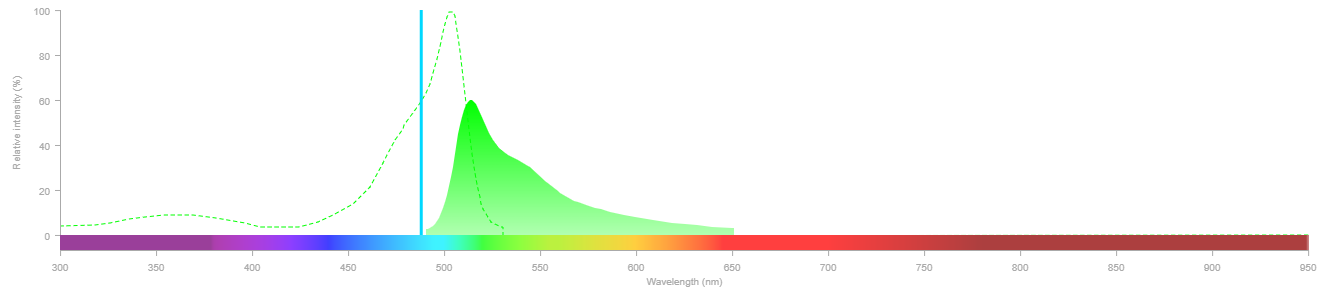
**

**Impact**

**Figure S1**: Fluorescence spectra of **green** fluorescently labelled 30 nm polystyrene nanoplastics, excitation 480 nm and emission at 500 nm


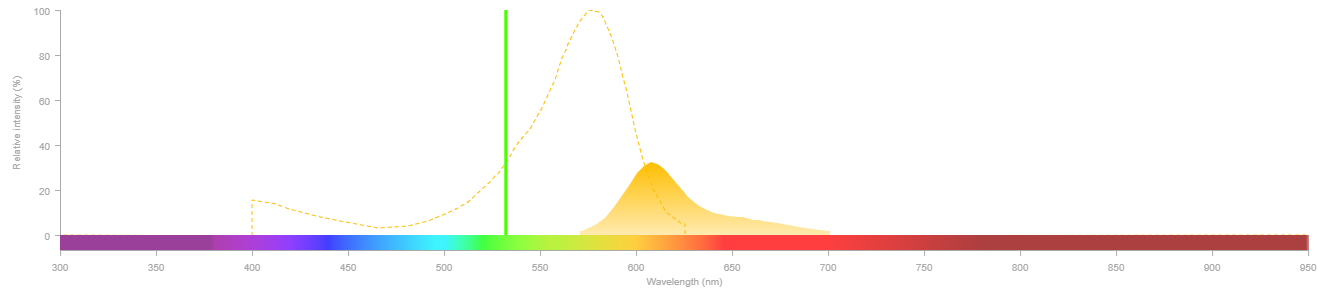


**Figure S2**: Fluorescence spectra of **red** fluorescently labelled 20 nm polystyrene nanoplastics, excitation at 580 nm and emission at 605 nm.


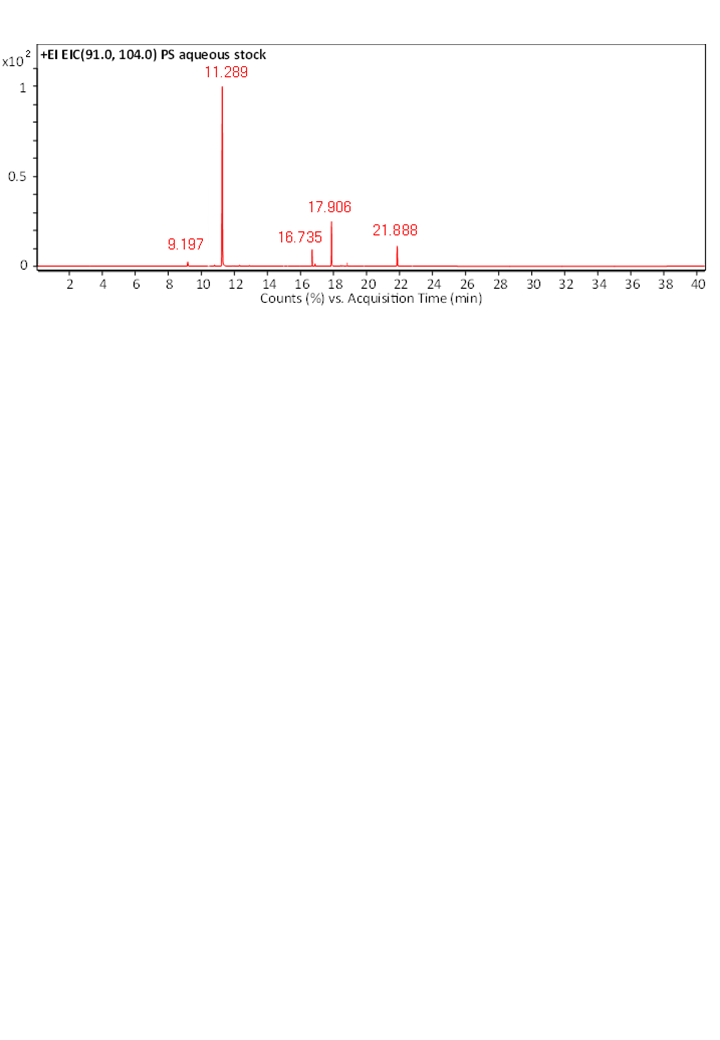


**Figure S3**: Extracted ion chromatogram (m/z 91, 104) of 30 nm polystyrene nanoplastics. The chromatogram shows peaks corresponding to specific pyrolysis products of polystyrene. The highest peak on the chromatogram corresponds to styrene, at retention time 11.289 minutes. Peaks at retention times 16.735, 17.906 and 21.888 minutes correspond to dibenzyl, styrene dimer and styrene trimer, respectively.


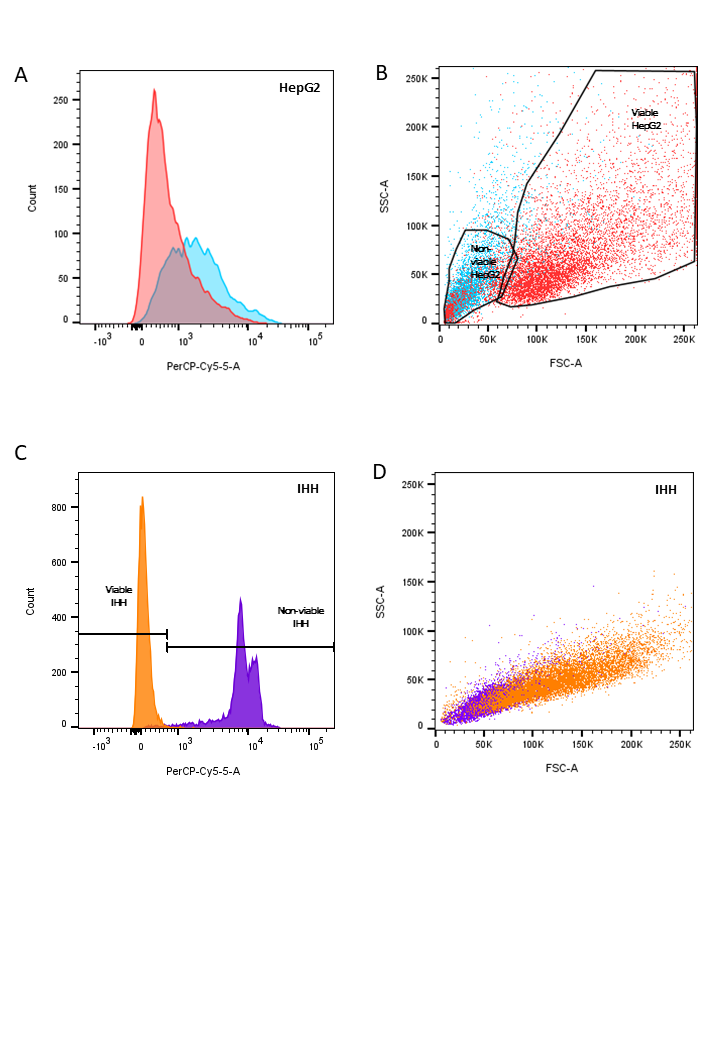


**Figure S4**: Gating strategy for cell viability analysis in IHH and HepG2 cells. A) No distinct difference was seen in the PerCP-Cy5 signal of non-viable (blue) and control (red) HepG2 cells after 7-AAD staining. B) The scatter plot shows two distinct populations of viable (red) and non-viable (blue) HepG2 cells. C) A clear difference was seen in the PerCP-Cy5 signal between viable (orange) and non-viable (purple) IHH cells after 7-AAD staining. D) The scatter plot shows viable (orange) and non-viable (purple) IHH cell populations.


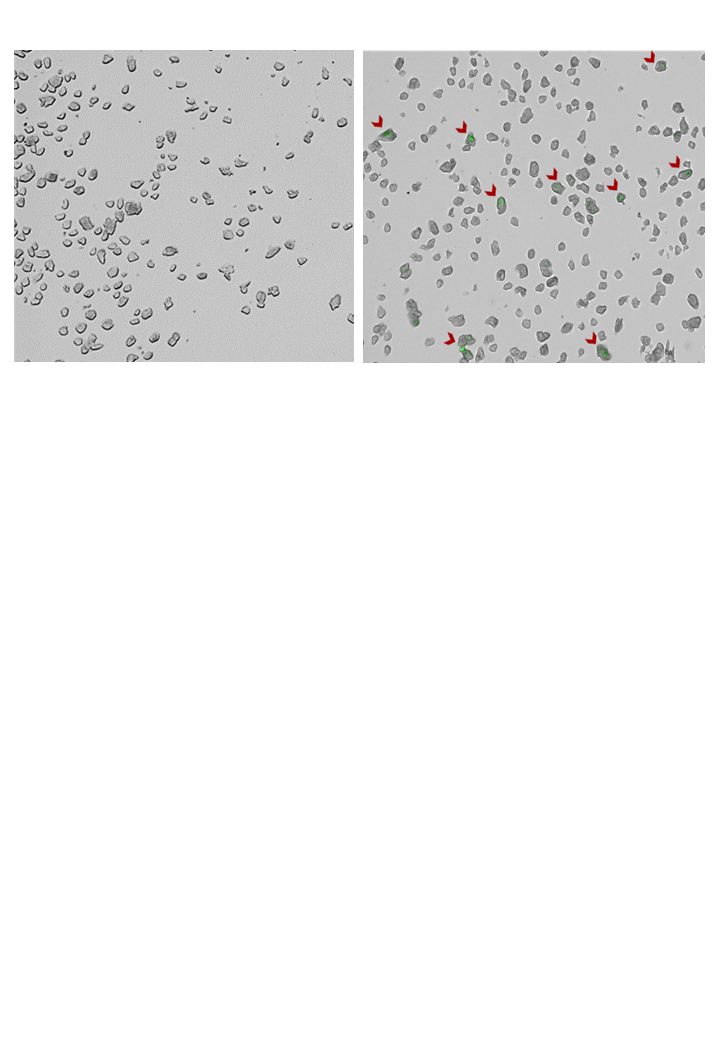


**Figure S5**: Fluorescence microscopy images of cells isolated from liver slices showing PS NPs uptake in hPCLS.


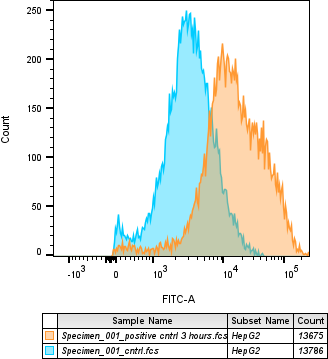


**Figure S6**: 2.5 mg/mL FeSO_4_.10H2O was used as a positive control (orange) for DCFDA assay and gave a distinct FITC signal compared to the control (blue) HepG2 cells.

| **S. No** | **Conditions** | **Details** |
| --- | --- | --- |
| 1 | Cell Density | 30,000 cells/well |
| 2 | Baseline | Cycles: 5  Mix: 3 mins  Wait: 0 mins  Measure: 3 mins |
| 3 | Oligomycin | Concentration used: 2.5 µM  Cycles: 3  Mix: 3 mins  Wait: 2 mins  Measure: 3 mins |
| 4 | FCCP | Concentration used: 2 µM  Cycles: 4  Mix: 3 mins  Wait: 2 mins  Measure: 3 mins |
| 5 | Rotenone/Antimycin A | Concentration used: 0.5 µM  Cycles: 3  Mix: 3 mins  Wait: 2 mins  Measure: 3 mins |

**Table S1**: Inhibitors/Uncoupler concentrations and measurement details for the Seahorse XFe96 assay

**
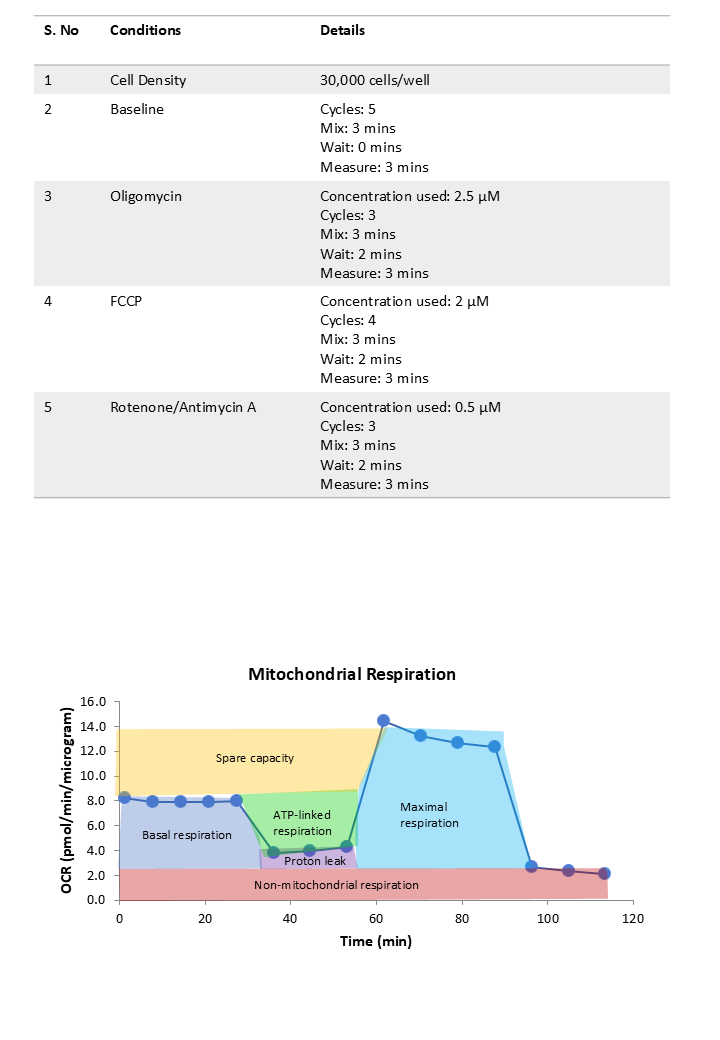
**

**Figure S7**: Seahorse XF Cell mito stress test profile, showing key parameters for mitochondrial function (adapted from User guide Part no. 103015-100)

|  | **SEX** | **AGE** | **ETHNICITY** | **BMI** | **AETILOGY** | **TREATMENT (Y/N)** |
| --- | --- | --- | --- | --- | --- | --- |
| **ILB129** | M | 78 | White british | 24.3 | CRLM | Y |
| **ILB130** | F | 57 | White british | 28.1 | CRLM | Y |
| **ILB134** | F | 64 | White british | 31.8 | CRLM | N |
| **ILB138** | M | 62 | White british | 29.6 | CRLM | Y |
| **ILB174** | F | 58 | White British | 21.5 | CRLM | Y |

**Table S2**: Baseline characteristics of liver tissue donors for precision-cut liver slices preparation.

Abbreviations: BMI- Body mass index; CRLM- colorectal liver metastasis.
